# *Anopheles parensis* contributes to residual malaria transmission in South Africa

**DOI:** 10.1186/s12936-019-2889-5

**Published:** 2019-07-29

**Authors:** Ashley Burke, Yael Dahan-Moss, Frances Duncan, Bheki Qwabe, Maureen Coetzee, Lizette Koekemoer, Basil Brooke

**Affiliations:** 1 Wits Research Institute for Malaria and Wits/MRC Collaborating Centre for Multidisciplinary Research On Malaria, School of Pathology, Faculty of Health Sciences, University of the Witwatersrand, Johannesburg, South Africa; 2 Centre for Emerging Zoonotic and Parasitic Diseases, National Institute for Communicable Diseases, Johannesburg, South Africa; 3 School of Animal, Plant & Environmental Sciences, University of the Witwatersrand, Johannesburg, South Africa; 4 Environmental Health, Malaria and Communicable Disease Control, KwaZulu-Natal Department of Health, Jozini, South Africa

**Keywords:** *Anopheles parensis*, Secondary vector, *Plasmodium falciparum*, Residual malaria, Incrimination

## Abstract

**Background:**

Understanding the contribution of outdoor-resting *Anopheles* mosquitoes to residual malaria transmission is important in terms of scaling up vector control towards malaria elimination in South Africa. The aim of this project was to assess the potential role of *Anopheles parensis* and other *Anopheles* species in residual malaria transmission, using sentinel surveillance sites in the uMkhanyakude District of northern KwaZulu-Natal Province.

**Methods:**

Monthly vector surveillance was conducted at the sentinel sites from January 2017 to May 2018. Outdoor-placed clay pot resting traps were used to collect male and female adult *Anopheles* mosquitoes. All *Anopheles gambiae* complex and *Anopheles funestus* group specimens collected were identified to species and all females were screened for *Plasmodium falciparum* circumsporozoite protein (CSP) by enzyme-linked immunosorbent assay (ELISA). Samples showing infectivity for *P. falciparum* were further verified by a nested PCR and subsequent DNA sequence analysis.

**Results:**

From a sample of 491 anophelines, *Anopheles arabiensis* (*n* = 228) and *An. parensis* (*n* = 194) were the most abundant. Other species collected included *Anopheles merus* (*n *=11), *Anopheles quadriannulatus* (*n *= 10)*, Anopheles leesoni* (*n *= 29), *Anopheles rivulorum* (*n *=18), and *Anopheles vaneedeni* (*n *=1). Of the 317 female specimens screened for *P. falciparum* CSP, one *Anopheles arabiensis* and one *An. parensis* showed positive by ELISA and *Plasmodium* nested PCR. For the *An. parensis* specimen, confirmation of its species identity was based on sequence analysis of the ITS2 region, and the presence of *P. falciparum* DNA was further confirmed by sequence analysis.

**Conclusions:**

*Anopheles parensis* is a potential vector of malaria in South Africa although its contribution to transmission is likely to be minimal at best owing to its strong zoophilic tendency. By contrast, *An. arabiensis* is a major vector that is primarily responsible for the bulk of residual malaria transmission in South Africa. As all recently collected sporozoite-positive *Anopheles* mosquitoes were found in outdoor-placed resting traps, it is necessary to introduce interventions that can be used to control outdoor-resting vector populations while maintaining the efficacy of South Africa’s indoor house spraying operations.

## Background

Malaria in South Africa is endemic in the low altitude northeastern border regions of KwaZulu-Natal, Mpumalanga and Limpopo provinces, and transmission follows a seasonal trend with peaks during the rainy season of November to April. Almost all infections are *Plasmodium falciparum* [1]. Malaria vector control operations in the affected provinces primarily utilize insecticide-based indoor residual spraying (IRS) with supplementary larviciding in select districts where incidence is highest. These interventions have reduced overall incidence to a point where elimination is a feasible prospect, leading to the development of an elimination strategy [2]. There was however an unexpected and substantial increase in cases in 2017 throughout the southern African region [3]. This increase coupled with ongoing residual transmission, despite control operations in South Africa, has necessitated intensified vector surveillance activities designed to better understand the entomological drivers of transmission [4].

The primary vectors of malaria in South Africa are *Anopheles funestus* and *Anopheles arabiensis* [5]. The former species is highly anthropophilic and endophilic, making it especially susceptible to control by IRS. As a result of South Africa’s IRS operations, the incidence of *An. funestus* has been reduced to a point where it is almost entirely undetectable using a range of surveillance methods [6]. *Anopheles arabiensis* on the other hand shows variable feeding and resting habits, and will readily rest outdoors making it less amenable to control by IRS. This species has recently been directly incriminated in malaria transmission in South Africa [7] and is considered to be the primary contributor to residual incidence. Added to this list is *Anopheles merus*, which has been directly implicated in transmission in southern Mozambique [8] and which occurs in South Africa in all malarious provinces [9, 10]. *Anopheles vaneedeni*, an outdoor resting member of the *An. funestus* group [11], has recently been incriminated as a secondary vector of malaria in South Africa and a likely contributor to residual transmission [12].

Members of the *An. funestus* group that have been incriminated in malaria transmission in other African countries are *Anopheles rivulorum, Anopheles leesoni* and *Anopheles parensis* (tentative) in Tanzania [13–15], *Anopheles rivulorum* in Kenya [16], and *Anopheles longipalpis* in Kenya [17]. *Anopheles parensis* periodically appears as a potential vector. This species is predominantly zoophilic and rarely takes blood from humans even though some populations have shown a strong inclination to rest indoors [18, 19]. A single positive *An. parensis* (1 out of 4 specimens sampled) using nested PCR was detected in Bagamoya, Tanzania [14]. Samples of *An. parensis* from Uganda gave a *P. falciparum* infection rate of 4.2% (n = 94) using a Taqman assay but this was not confirmed by nested PCR, suggesting the detection of false positives [20]. Tentative evidence of malaria infectivity by this species was obtained from northern KwaZulu-Natal Province in South Africa based on a sample of 149 specimens of which 20 (13.4%) showed positive using the standard ELISA assay [19] for *P. falciparum* circumsporozoite protein. Follow-up PCR analysis however did not reveal any positives and it was subsequently shown that all ELISA positive specimens had taken blood from non-human sources [19]. The status of *An. parensis* as a malaria vector, therefore, remains to be confirmed.

Understanding the contribution of outdoor-resting anophelines to residual malaria transmission is important in terms of scaling up vector control toward malaria elimination in South Africa. The aim of this project was therefore to assess the potential role of *An. parensis* and other *Anopheles* species in residual malaria transmission using sentinel sites in the uMkhanyakude District of northern KwaZulu-Natal Province, which currently experiences very low malaria incidence and is the closest of all of South Africa’s malaria-endemic districts to achieving elimination status.

## Methods

### Entomological surveillance

**Fig. 1:**
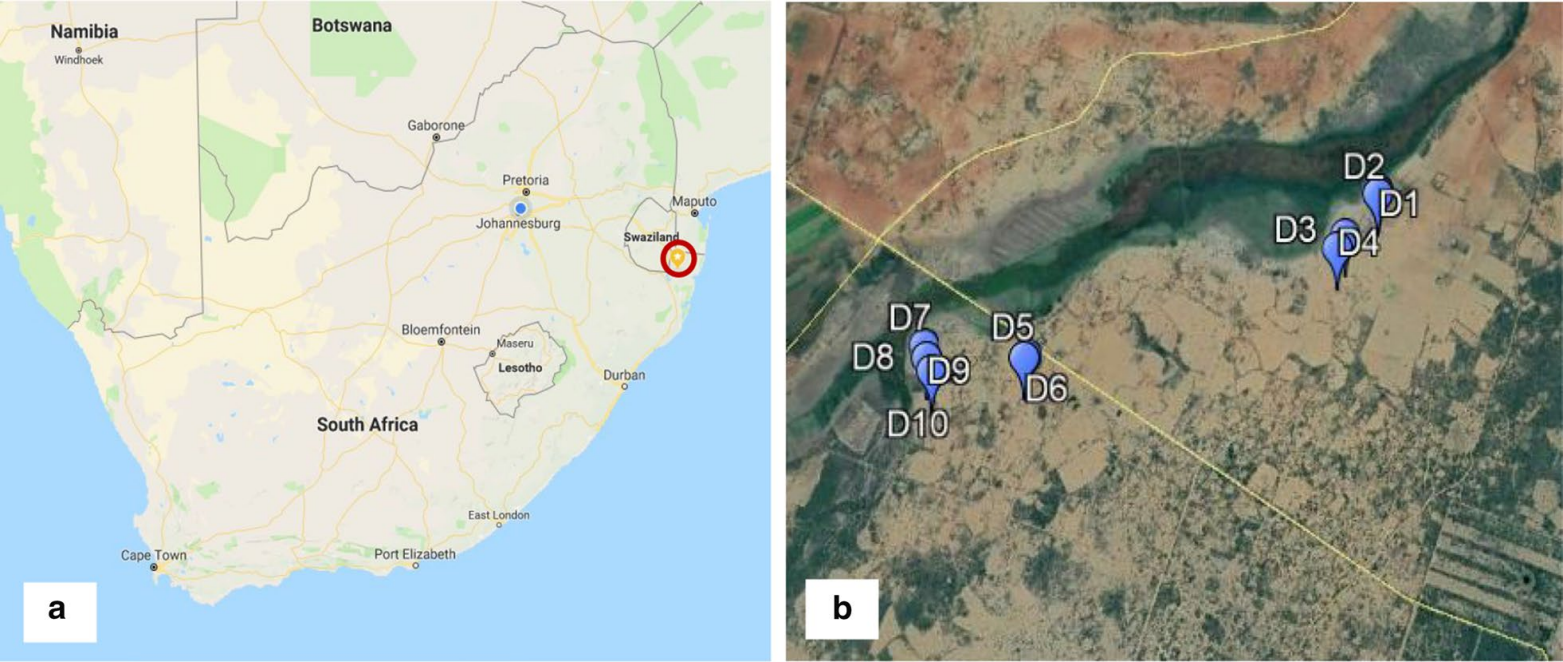
**a** Location of the field site at Mamfene (yellow pin circled in red), KwaZulu-Natal Province, South Africa. Map data © 2018 AfriGIS (Pty) Ltd, Google. **b** Spatial distribution of 10 clay pots (blue pins) deployed as outdoor-placed resting traps at the field site in KwaZulu-Natal Province. Image © 2018 DigitalGlobe, Google Earth © Google 2018

**Fig. 2:**
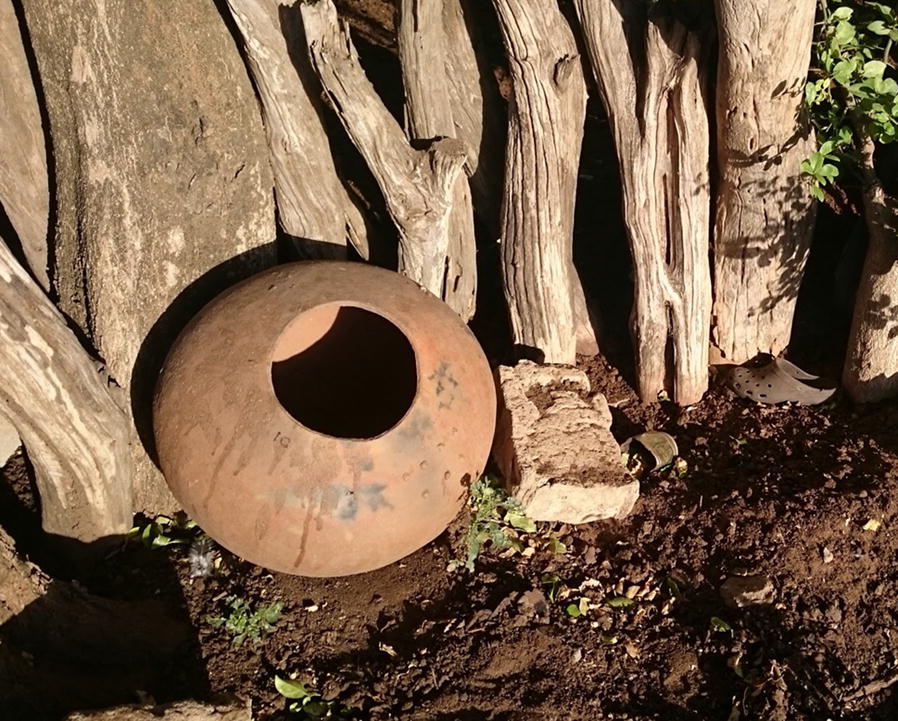
An example of the locally-sourced clay pots that were used as outdoor-placed resting traps for *Anopheles* mosquito collections in KwaZulu-Natal Province, South Africa

Monthly entomological surveillance was conducted for just over 1 year—January 2017 to May 2018—at sentinel sites in the rural village of Mamfene, in the uMkhanyakude District of KwaZulu-Natal Province, South Africa (Fig. 1). Traditional clay pots (Fig. 2) were deployed outside households (with the home-owners’ consent) to serve as resting traps from which live *Anopheles* mosquitoes were collected in the cool early hours of each morning (between 06:00 and 08:00). Five households were recruited for this purpose and two pots were deployed in each (n = 10 pots). All pots were cleared of mosquitoes for 3 days each month during the surveillance period, except for June 2017 and February 2018. There were no collections during those two months owing to logistical constraints.

### Molecular identification

All specimens were identified by external morphology using dichotomous keys [21]. Those identified as *Anopheles gambiae* complex or *An. funestus* group were dry-preserved on silica gel and transported to the Vector Control Reference Laboratory of the National Institute for Communicable Diseases in Johannesburg for further processing. Each specimen was identified to species using the appropriate polymerase chain reaction (PCR) protocol for either *An. gambiae* complex [22] or *An. funestus* group [23, 24].

### *Plasmodium* infectivity testing

Every female anopheline mosquito was screened for the presence of *P. falciparum* circumsporozoite protein (CSP) by means of an enzyme-linked immunosorbent assay (ELISA) [25, 26]. The ELISA homogenates of all positive specimens were then boiled at 100 °C for 10 min followed by a repeat ELISA to confirm or refute the presence of CSP [27]. Those that remained ELISA-positive were verified by a nested *Plasmodium* PCR assay [28]. The internal transcribed spacer 2 (ITS2) region of an anopheline female showing positive for a *P. falciparum* infection was amplified by ITS2 PCR assay [23] to be used for DNA sequence analysis to verify the species identification.

### DNA sequence analysis

Sequencing analysis of the ITS2 and the nested *Plasmodium* PCR products was used to confirm the anopheline species identity and infection with *P. falciparum*. Purification and sequencing of the PCR products was performed by Macrogen. Subsequently, the chromatograms of the sequences were manually edited by BioEdit version 7.2.5 [39]. The Emboss Needle pairwise sequence alignment tool (https://www.ebi.ac.uk/Tools/psa/emboss_needle/nucleotide.html) was used to compare the analysed *An. parensis* ITS2 sequence with the established *An. parensis* ITS2 reference sequences (GenBank accession number JN994144.1) [29]. The unknown *Plasmodium* sequence was compared with the reference *P. falciparum* Pf8 18S ribosomal RNA gene, partial sequence (GenBank accession number: KC428742.1) [30].

## Results

### Entomological surveillance

**Fig. 3:**
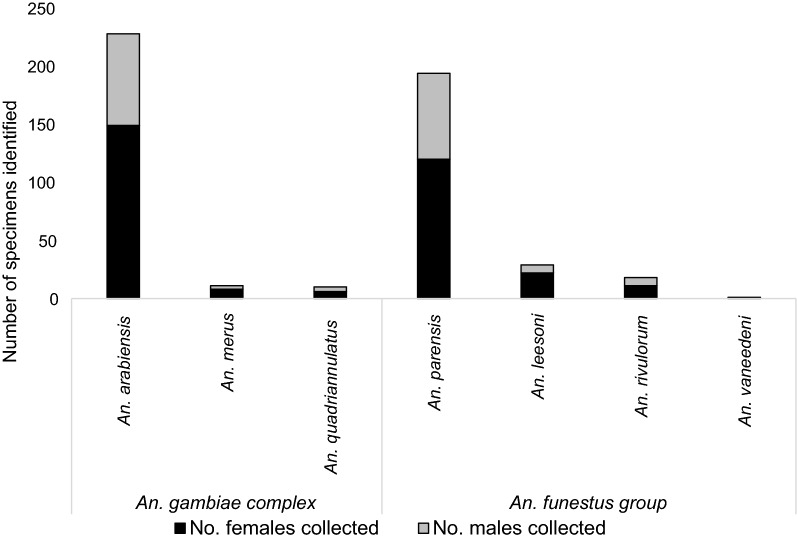
*Anopheles* mosquitoes by species group and gender collected using outdoor-placed clay pots deployed as resting traps at a field site at Mamfene, KwaZulu-Natal Province, South Africa, from January 2017 to May 2018

**Fig. 4:**
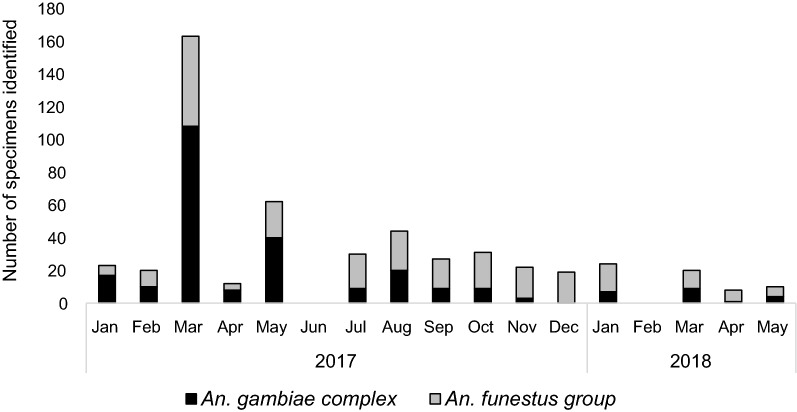
Monthly sampling of male and female *Anopheles* by species group/complex in Mamfene, KwaZulu-Natal Province, South Africa, from January 2017 to May 2018. Mosquitoes were collected over a 3-day period each month. No mosquitoes were collected for June 2017 and February 2018 due to logistical constraints

Adult *Anopheles* mosquitoes were collected from outdoor-resting traps deployed around households in Mamfene, northern KwaZulu-Natal Province (Figs. 1 and 2), during the period January 2017 to May 2018. A total of 491 male and female specimens were identified as either *An. gambiae* complex or *An. funestus* group using morphological features [21]. These were subsequently identified to species using the standard PCR assays for each group [22–24]. *Anopheles arabiensis,* a member of the *An. gambiae* complex, was the most abundant (*n* = 228). Other members of the *An. gambiae* complex included *An. merus* (*n* = 11) and *An. quadriannulatus* (*n* = 10) (Fig. 3). Within the *An. funestus* group, *An. parensis* was most abundant (n = 194) followed by *An. leesoni* (*n* = 29), *An. rivulorum* (*n* = 18) and *An. vaneedeni (n* = 1) (Fig. 3). There was an overall preponderance of females across all species collected (F = 240.58, df = 1, *P* < 0.004); 163 females and 86 males of the *An. gambiae* complex, and 154 females and 88 males of the *An. funestus* group (Fig. 3). The monthly and seasonal distribution of collected anophelines showed a significant increase in mosquito numbers in March 2017 (Fig. 4.). This increase in relative mosquito population density is likely attributable to high rainfall experienced in Mamfene from February to March 2017.

### Vector incrimination

All female mosquitoes were screened for *P. falciparum* CSP by a two-step ELISA [25–27] and were subsequently validated by nested PCR and DNA sequence analysis where necessary. The majority of positive samples from the first ELISA assay did not appear positive on the second that included a boiling step. Of the 163 female *An. gambiae* complex specimens screened for CSP, one *An. arabiensis* (collected in October 2017) showed positive by ELISA and *Plasmodium* nested PCR [28]. As this *An. arabiensis* population has been implicated in malaria transmission before [7], no further DNA sequence analysis was considered necessary. Of the 154 female *An. funestus* group specimens screened, one female identified as *An. parensis* (also collected in October 2017) showed positive for *P. falciparum* CSP by ELISA and nested *Plasmodium* PCR. These data indicate a *P. falciparum* infectivity rate of 0.67% (n = 149) for *An. arabiensis* and 0.83% (n = 120) for *An. parensis* at Mamfene.

### Validation of *Anopheles* species identity and the presence of a *Plasmodium* infection

The species identity of the infective *An. parensis* female was confirmed after standard PCR [23] ITS2 sequences showed 99% homology between this specimen and that for a reference sequence of this species in GenBank (accession number: JN994144.1) [25]. The *P. falciparum* infection in this *An. parensis* specimen was confirmed by 95% sequence homology of the nested *Plasmodium* PCR product with the *P. falciparum* isolate Pf8 18S ribosomal RNA gene, partial sequence (GenBank accession number: KC428742.1) [30].

## Discussion

The morphological and molecular taxonomy of the *An. funestus* group is comparatively complex [11], making identification of member species potentially problematic. The need for morphological identification prior to molecular analysis has been highlighted [31], and is especially critical in terms of follow-on vector incrimination. This is because *Anopheles* species misidentification can easily occur when entirely reliant on nested PCR assays, and sequence information can be misleading in terms of the critical thresholds required for conspecific homology between unknown samples and banked sequences linked to voucher specimens. In addition to the complexities of *Anopheles* species identification, establishing *Plasmodium* infectivity (as distinct from infection) also requires careful use of methodologies and data interpretation. It is for this reason that the two-step ELISA assays for CSP detection were conducted prior to molecular verification for the *An. parensis* samples. Given that *An. parensis* has not been unambiguously incriminated in malaria transmission before, it was considered necessary to ensure that all morphological, PCR, ELISA and sequence data were sufficiently aligned to confirm *Plasmodium* infectivity, and that all data were either double or triple-checked for quality assurance. A similar approach was recently conducted for the incrimination of *An. vaneedeni* (also a member of the *An. funestus* group) as a secondary malaria vector in South Africa [12].

It should be noted that the single ELISA technique used to detect *Plasmodium* sporozoites can lack sensitivity and specificity. It is however more likely to give a false positive than a false negative result, especially in zoophilic species such as *An. parensis* [19, 27]. The false positive ELISA result that can occur in zoophilic *Anopheles* species is due to a cross-reacting heat-unstable antigen [27]. Heating the ELISA lysate, as was done in the experiments described here, reduces the possibility of obtaining false positives. Additionally, the use of nested PCR for the detection of *Plasmodium* sporozoites is a highly sensitive method [28] that further eliminates the chance of false positives. Although Taqman assays [32, 33] are also effective for detecting *Plasmodium* in anophelines, they can be prohibitively expensive. Owing to South Africa’s comparatively low malaria incidence and the general rarity of *Plasmodium* infective mosquitoes, the two-step ELISA method followed by nested PCR is the most cost-effective and ideal method for detecting and confirming *Plasmodium* infectivity.

It is reasonable to infer that the single *An. parensis* female that presented with *P. falciparum* CSP was infective for malaria. This at least shows that this species is a potential malaria vector. There are however other considerations that need to be taken into account in terms of its contribution to malaria transmission. Available data show that *An. parensis* seldom takes blood from humans, being more inclined to feed on livestock animals and rest outdoors [34, 35], as has previously been demonstrated for the northern KwaZulu-Natal population [19]. This reduces its potential contribution to malaria transmission substantially, but not necessarily to zero. This is reinforced by earlier records that describe a high malaria incidence setting on the Kenyan coast in which *An. parensis* was abundant but no sporozoite positive specimens were found (based on salivary gland dissections) despite the sympatric *An. funestus* population showing a sporozoite positive index of 7% [35]. *Anopheles parensis* has nevertheless been recorded as an exophilic feeder on humans [35] with a comparatively high human blood index in some ecosystems [34, 36], and can be found resting both indoors and outdoors [37].

*Anopheles arabiensis* is a major malaria vector throughout its distribution including northern KwaZulu-Natal [7, 38]. It is worth noting that both the infective *An. arabiensis* and *An. parensis* specimens were collected in October 2017. Although October is generally a low-incidence month in South Africa, 2017 was an unusual year in that unexpectedly high numbers of cases were recorded from May onwards (unpublished National Department of Health statistics). Incidence in KwaZulu-Natal was nevertheless low compared to other endemic provinces which precludes associating these sporozoite-positive specimens with any case or cluster of cases. Also important to note is that both of these specimens were caught resting outdoors, highlighting the importance of addressing residual transmission in South Africa by targeting outdoor-resting vectors in addition to those targeted indoors by the provincial IRS programmes. Intensive provincial larval source management programmes, including winter larviciding, and community outreach programmes designed to educate on personal protection measures and treatment-seeking, are currently under development to address this issue.

## Conclusions

It is concluded that *An. parensis* is a potential vector of malaria but that its contribution to transmission in South Africa’s endemic provinces is likely to be minimal at best owing to its strong zoophilic tendency [19]. By contrast, *An. arabiensis* is a major vector throughout its distribution and is primarily responsible for the bulk of residual malaria transmission in South Africa. As all recently collected sporozoite-positive *Anopheles* mosquitoes were found in outdoor-placed resting traps in northern KwaZulu-Natal Province [7, 12], it is necessary to introduce additional interventions, intensive larval source management in particular, that can be used to control outdoor-resting vector populations while maintaining the efficacy of South Africa’s IRS operations for the ongoing control of indoor-resting vectors.

## Data Availability

The datasets used and/or analysed during the current study are available from the corresponding author on reasonable request. All data presented in this study is stored in the malaria vector database of the Vector Control Reference Laboratory, National Institute for Communicable Diseases, and is available on request.

## Abbreviations

CSP: circumsporozoite protein
DNA: deoxyribonucleic acid
ELISA: enzyme-linked immunosorbent assay
IRS: indoor residual spraying
ITS2: internal transcribed spacer 2
PCR: polymerase chain reaction
RNA: ribonucleic acid
